# *Mecp2* Deficiency in Peripheral Sensory Neuron Improves Cognitive Function by Enhancing Hippocampal Dendritic Spine Densities in Mice

**DOI:** 10.3390/cells13110988

**Published:** 2024-06-06

**Authors:** Yuting Feng, Jingge Wang, Jun Liu, Yinwei Zhou, Ying Jiang, Wenhui Zhou, Feng Wu, Xingjun Liu, Lin Luo

**Affiliations:** School of Pharmacy, Nantong University, Nantong 226001, China; 1727021041@stmail.ntu.edu.cn (Y.F.); 2119310027@stmail.ntu.edu.cn (J.W.); jliu@innostar.cn (J.L.); 15371149801@163.com (Y.Z.); 1827021019@stmail.ntu.edu.cn (Y.J.); 2119320006@stmail.ntu.edu.cn (W.Z.); wf619@ntu.edu.cn (F.W.)

**Keywords:** methyl-CpG-binding protein 2, peripheral sensory neuron, cognitive function, BDNF/CREB1 pathway

## Abstract

Methyl-CpG-binding protein 2 (*Mecp2*) is an epigenetic modulator and numerous studies have explored its impact on the central nervous system manifestations. However, little attention has been given to its potential contributions to the peripheral nervous system (PNS). To investigate the regulation of *Mecp2* in the PNS on specific central regions, we generated *Mecp2^fl/fl^Advillin^cre^* mice with the sensory-neuron-specific deletion of the *Mecp2* gene and found the mutant mice had a heightened sensitivity to temperature, which, however, did not affect the sense of motion, social behaviors, and anxiety-like behavior. Notably, in comparison to *Mecp2^fl/fl^* mice, *Mecp2^fl/fl^Advillin^cre^* mice exhibited improved learning and memory abilities. The levels of hippocampal synaptophysin and PSD95 proteins were higher in *Mecp2^fl/fl^Advillin^cre^* mice than in *Mecp2^fl/fl^* mice. Golgi staining revealed a significant increase in total spine density, and dendritic arborization in the hippocampal pyramidal neurons of *Mecp2^fl/fl^Advillin^cre^* mice compared to *Mecp2^fl/fl^* mice. In addition, the activation of the BDNF-TrkB-CREB1 pathway was observed in the hippocampus and spinal cord of *Mecp2^fl/fl^Advillin^cre^* mice. Intriguingly, the hippocampal BDNF/CREB1 signaling pathway in mutant mice was initiated within 5 days after birth. Our findings suggest a potential therapeutic strategy targeting the BDNF-TrkB-CREB1 signaling pathway and peripheral somasensory neurons to treat learning and cognitive deficits associated with Mecp2 disorders.

## 1. Introduction

DNA methylation, as an important epigenetic modification, plays a crucial role in the development and maintenance of the central nervous system [1]. It is found that methyl-CpG-binding protein 2 (*Mecp2*) binds to methylated CpG (mCG) via its methyl-binding domain (MBD) and is mainly involved in the inhibition and regulation of gene expression in neuronal cells and recruits co-inhibitors and chromatin remodeling proteins by binding to these methylated CpG sites to achieve transcriptional inhibition [2]. Studies in patients and mouse models demonstrate that both the loss and gain of *Mecp2* function can result in abnormal social behavior. The X-linked gene encoding *Mecp2* is associated with two serious and complex neurodevelopmental disorders. One is *Mecp2* mutations which is the major cause of Rett syndrome (RTT) [3]. The other is *Mecp2* duplication syndrome (MDS). The symptoms of both diseases are characterized by motor disabilities, autism or autistic features, and seizures [4,5]. These bidirectional associations indicate the fundamental role of *Mecp2* in the brain and demonstrate the importance of precisely controlled *Mecp2* expression for normal development and neuronal function [6]. Due to the extensive binding pattern of *Mecp2* and the inherent neuronal heterogeneity, various *Mecp2* mutations result in distinct effects on gene expression within diverse cell types [7]. Therefore, the molecular effects of *Mecp2* are still elusive so far, as the functional changes caused by the variation in its gene are mostly subtle.

It is widely accepted that RTT or MDS are caused by an *Mecp2* function disorder in the central nervous system (CNS) with relatively little contribution coming from its roles in peripheral organs as observed in *Mecp2*-deficiency mice [8]. Thus, the predominant focus of *Mecp2* research has focused on the CNS manifestations of *Mecp2*-related disorders and their neural underpinnings, with little attention given to its potential contributions to the peripheral nervous system (PNS). But recent evidence indicates a connection between *Mecp2* and peripheral somasensory functions. Findings from two large datasets [9,10] indicated that approximately two-thirds of individuals with RTT exhibit a reduced sensitivity to pain [11]. Additionally, certain animal studies have further established the associations between *Mecp2* function and pain sensitivity [12]. Furthermore, children with autism-spectrum disorder (ASD) often exhibit tactile discrimination and hypersensitivity to gentle touch [13], which were also observed in ASD mice and the mechanism was relevant to the deletion of *Mecp2* in peripheral mechanosensory neurons [14]. It indicates that *Mecp2* can directly regulate PNS function; yet, variations exist in how *Mecp2* impacts different somatosensory neurons in the PNS. Thus far, it is unclear whether *Mecp2* deficiency in sensory neurons is confined solely to impairments in the sense of touch and whether these deviant somatosensory responses contribute to region-specific abnormal brain development and exert effects on cognitive function.

It has been found that *Mecp2* in CNS plays an important role in synaptic formation and maintenance, which is crucial for synaptic plasticity. Studies conducted on transgenic mice and patients with RTT or MDS have not only revealed synaptic dysfunction in the hippocampus and cortex but also observed abnormalities in dendritic growth and morphological alterations. The deficiency of *Mecp2* in the CNS induced atypical structural synaptic plasticity, a reduction in paired-pulse responses, and alterations in various parameters of short-term synaptic plasticity. The cerebral cortex of MDS mice showed an abnormal increase in the formation and stability of dendritic spines [15]. Moreover, *Mecp2* can ameliorate age-related cognitive decline by modulating synaptic plasticity through the activation of the Ca^2+^/cyclic AMP response element-binding protein 1 (CREB1) pathway [16]. Certain neurotrophic factor signaling pathways, such as brain-derived neurotrophic factor (BDNF), and neurotrophic factors 3 (NT3) and 4 (NT4), are thought to play a role in the Mecp2-mediated enhancement of synaptic strength and neurotransmission [17]. Qiao et al. found that BDNF was synthesized by primary afferent neurons located in the dorsal root ganglia (DRG) in rats, and released to spinal nerve terminals in response to depolarization [18]. In addition, a recent work of Orefice et al. found that the loss of *Mecp2* in peripheral somatosensory neurons can lead to some alterations in the neurochemical substances input to CNS and change the functional characteristics of brain circuits [13]. But the regulatory effects of *Mecp2* in the peripheral sensory neurons on specific central regions and the underlying mechanisms remain unclear.

In this study, we employed mice with a conditional knockout of *Mecp2* in peripheral sensory neurons, along with behavioral testing and dendritic spine analysis, to assess the influence of *Mecp2* in peripheral nervous system (PNS) neurons on cognitive function. Our findings may reveal the neurobiological mechanisms of aberrant sensory responses leading to region-specific alterations in brain function.

## 2. Materials and Methods

### 2.1. Materials and Reagents

Antibodies against Mecp2 (Cat# 3456S) and PSD95 (Cat# P78352) were obtained from Cell Signaling Technology (Danvers, MA, USA). HRP-conjugated AffiniPure goat anti-rabbit and anti-mouse IgG(H+L) (Cat# SA00001-2 and SA00001-1), alpha- tubulin monoclonal antibody (Cat# 66031-1-Ig), TrkB (Cat# 13129-1-AP), brain-derived neurotrophic factor (BDNF) (Cat# 28205-1-AP), CREB1 (Cat# 12208-1-AP), and phospho-CREB1 (Ser133) (Cat# 28792-1-AP) rabbit polyclonal antibody were purchased from Proteintech (Rosemont, USA). Anti-synaptophysin antibody (Cat# ab16659) was from Abcam (Cambridge, UK). Anti-GAPDH (Cat# GB11002) and anti-beta actin (Cat# GB11001) antibodies were obtained from Servicebio Biotechnology Co., Ltd. (Wuhan, China).

### 2.2. Mecp2^fl/fl^Advillin^cre^ Mice Breeding

*Mecp2^fl/fl^* mice (011918) were obtained from Mutant Mouse Regional Resource Center at University of California Davis [19]. *Advillin^cre^* mice (032536) were from Jackson Laboratory (Bar Harbor, ME, USA). All mice with a pure genetic background (*C57BL/6J*) were used for behavioral analyses. *Mecp2* floxed mice and *Advillin-Cre* mice were housed in pathogen-free conditions. Female *Mecp2^fl/fl^* mice were bred with male *Advillin-Cre* mice to generate *Mecp2^fl/fl^Advillin^cre^* mice. The following PCR primers were used to distinguish between wild-type *Mecp2* and *Mecp2* floxed allele: Forward: 5′-caccacagaagtactatgatc-3′; and Reverse: 5′-ctaggtaagagctcttgttga-3′. The PCR primers to *Advillin-Cre* allele are: Forward: 5′-gcactgatttcgaccaggtt-3′; and Reverse: 5′-gagtcatccttagcgccgta-3′ [20]. All male mice were used at the age of 8 to 10 weeks (body weight: 22–25 g), and we randomly included 10 to 15 mice per group, as indicated in each experiment. All mice were kept at conditions with 12 h light/dark cycle (lights on from 07:00 to 19:00), 21 ± 1 °C ambient temperature, 50 ± 10% relative humidity, and free access to food and water. All experiments were conducted according to protocols approved by Nantong University Institutional Animal Care and Use Committee.

### 2.3. Sensory Analysis

#### 2.3.1. Cold-Water Tail-Flick Latency Reflex Test

The cold-water tail-flick test was used to evaluate the cold sensitivity [21]. Mice were placed in cylindrical, plexiglass tubes with their tails exposed at the rear of the tube for 15 min, after habituating for 2 days. The tail-flick latencies were determined by immersing one-half to two-thirds of the tail in a mixture of equal parts water and ice cooled to 0 °C for a maximum of 60 s. The end point of the test occurred when the mice flicked their tails out of the water or reached the 60 s cut-off time. Each mouse was tested 3 times (with a 30 min interval between each test), and the average tail-flick latencies were analyzed.

#### 2.3.2. Hot-Plate Test

The hot-plate test was performed to evaluate sensitivity to pain stimuli [22]. Each mouse was placed as gently as possible on a hot plate at a temperature of 55.0 ± 0.5 °C (Techman Instrument, Chengdu, China), and continuously exposed to this temperature until either hind paw exhibited a nociceptive response, which was defined as mouse licking either hind paw. The latency of the first paw response was recorded with a cut-off time of 60 s. Each mouse was tested twice (with a 10 min interval) and the average latency of the first paw response was analyzed.

### 2.4. Behavioral Analysis

All behavioral experiments were performed between 8 am and 5 pm. All mice were subjected to the behavioral tests at 8 weeks of age in the order shown in Supplementary Figure S1 (*Mecp2^fl/fl^*, *n* = 10; *Mecp2^fl/fl^Advillin^cre^*, *n* = 11–15). The behaviors of open field and Morris water maze were recorded and analyzed by a video-tracking system (ANYmaze, Stoelting Co., Wood Dale, IL, USA). All apparatuses were wiped with water first, then with 75% ethanol, and were allowed to dry before being placed in mice. All animals in this work were euthanized one week after completing all behavioral experiments to collect samples for immunohistochemical and biochemical analyses.

#### 2.4.1. Open Field Test

As described [23], each mouse was placed in a plastic arena (48 cm long × 48 cm wide × 40 cm high) (Xinruan Information Technology Co., Ltd., Shanghai, China) at the center region and allowed to travel freely in the chamber. The exploration of mice was tracked with a total time of 10 min. A 15 × 15 cm^2^ square at the center of the plastic arena was designated as the central zone. The total distance and the time of the mice exploring in the central zone were recorded and analyzed.

#### 2.4.2. Marble-Burying and Nestlet-Shredding Test

In the marble-burying test, 20 standard glass toy marbles (5 rows of 4 marbles, assorted styles and colors, 15 mm diameter, 5.2 g in weight) were arrayed on the surface of clean bedding into a cage (26 cm × 48 cm × 20 cm) with fitted filter-top covers. The unscented mouse bedding material was 5 cm deep. Each mouse was carefully placed into a corner of the cage as far from marbles as possible, and we allowed it to remain in the cage undisturbed for 30 min [24]. Two task scorers who were unaware of the genotype of the tested mice counted the number of marbles buried. Score a marble as buried if two-thirds of its surface area was covered by bedding.

In the nestlet-shredding test [24], a nestlet comprised of pulped cotton fiber (5 cm × 5 cm, 5 mm thick, ~2.5 g each) was placed on top of fresh, unscented mouse bedding 0.5 cm depth in each cage (19 cm × 29 cm × 13 cm) with a filter-top cover. Each mouse was placed in a cage with the nestlet and undisturbed for 90 min. The height of the remaining intact nestlet was determined after a test session.

#### 2.4.3. Three-Chamber Social Approach and Social Novelty Test

Mice sociability and social novelty were evaluated in a three-chambered box (Ugo Basile, Gemonio, Italy) with openings between the chambers (each compartment is 20 cm wide × 40 cm long × 22 cm high) [14]. The outside walls of the chamber were opaque, while the inner dividers were clear plexiglass walls (8 cm high × 5 cm wide). After a 5 min habituation period in the empty chamber, the test mouse (Central) was moved into the empty center chamber with partitions in place. A novel mouse (Social) was settled in a wire cage in the left chamber and an empty mesh cup was placed in the right chamber during the “sociability” session. The partitions were then lifted, and the test mouse was free to explore all three sections of the chamber for 10 min. During the “social novelty preference” session, the test mouse was moved into the empty center chamber with partitions in place. A second novel mouse (Novel) was placed under the empty cup, partitions were removed, and the test mouse was allowed to freely explore the chamber for another 10 min. The time spent in each of the three chambers during each test session was recorded for evaluation.

The chronic social defeat stress (CSDS) mice were used as the positive control and they were constructed according to the reference [25]. Briefly, the eligibility of aggressive *CD1* mouse was selected, which attacked for at least two consecutive days, and the latency of first attack was less than 90 s but longer than 5 s. Then, each *C57BL/6J* mouse was placed as “invaders” into aggressive *CD1* mouse cages for 10 min per day. After that, *C57BL/6J* mice and *CD1* mice were separated by plastic dividers with holes for the next 24 h during which the stressful sensory cues from the aggressors persisted. The stress period lasted for 10 days.

#### 2.4.4. Object Recognition Test

The object recognition test was performed in the same plexiglass arena used for open-field testing [26]. Each mouse was habituated daily in the apparatus for 10 min for 4 consecutive days to become familiar with the apparatus and surrounding environment. On the fifth day, two identical objects (Object 1 and 2) were symmetrically placed into the apparatus 12 cm away from the front wall and 10 cm away from the right/left wall. During the familiarization phase, the mouse was allowed to explore the objects freely for 5 min. After a 15 min delay in its home cage, the animal was reintroduced into the arena to perform the test. During the test phase, one object (Object 2) was replaced with a novel object (Object 3) at the exact location. The mouse was allowed to explore the two objects for another 5 min. The time the mouse explored the objects was recorded in both phases and the discrimination index (DI) of the objects [DI = (Object 3 interaction − Object 1 interaction)/total interaction with both objects] was calculated [23].

#### 2.4.5. Elevated Plus-Maze

The elevated plus-maze apparatus was 80 cm high and consisted of two open arms (30 × 5 cm) and two enclosed arms (30 × 5 × 20 cm) which were facing opposite each other and connected by an open central zone (5 × 5 cm, Xinruan Information Technology Co., Ltd., Shanghai, China) [27]. The mouse was placed in the central square facing a closed arm, and allowed to freely explore for 5 min. The time each mouse spent on the open arms and closed arms were recorded and the ratio between the two was calculated.

#### 2.4.6. Morris Water Maze (MWM) Task

All mice were tested in a tank with a diameter of 180 cm and a height of 60 cm, which was divided into four imaginary equal quadrants. A 13 cm-diameter platform (island) was submersed 1 cm below the water surface and placed in the center of the quadrant in southwest (III). All mice were subjected to a four-day training trial. In the trial, each mouse was trained to locate the platform in 90 s. Those who did not reach the platform within 90 s were gently led to the platform by the experimenter and was left on the platform for 20 s. The training was conducted 4 times per day, and, between each training, the interval was more than 30 min. Meanwhile, the latency to the platform of every mouse on each day was recorded. On day 5, a spatial probe test was conducted for 90 s without the escape platform. The numbers of entries into the target zone, the distance traveled until the first entry into the target zone, and the mean speed in the pool were measured to evaluate mouse spatial memory [28,29].

#### 2.4.7. Pole-Climbing Test

A 9 mm-diameter 75 cm wooden rod was used as the pole. Each mouse was placed on the top of the pole (7.5 cm from the top of the pole) facing head-up and the total time of the mouse taken to reach the base of the pole was recorded, including the time of flipping and climbing down. The maximum cut-off time for stopping the test was 120 s. Before the test, each mouse was trained three times per day for two consecutive days with the intervals exceeding 30 min between each training session [30].

#### 2.4.8. Grip Strength Test

Neuromuscular strength was measured by a grip strength machine (CCE, Bioseb, Valenciennes, France). Each mouse was allowed to grasp the test grid (10 × 16 cm) with its forelimbs or with both fore- and hindlimbs, and its tail was gently pulled. The grip strength was expressed as gravity on the screen of the machine and the peak holding strength was recorded as force in grams [31].

### 2.5. Western Blot Analysis

Mouse dorsal root ganglia (DRG), spinal cord, and specific brain tissues (*n* ≥ 3) were homogenized in a 9-fold volume of ice-cold lysis buffer (50 mM Tris-HCl, pH 7.4, 2.0 mM EDTA, 8.5% sucrose, and 10 mM β-mercaptoethanol) containing a protease inhibitor tablet (Thermo Fisher Scientific, Waltham, MA, USA, Cat# a32963). The protein concentration was evaluated by BCA Protein Assay Kit (Thermo Fisher Scientific, Waltham, MA, USA, Cat# 23227). According to the standard protocol [32], all samples were separated in SDS-PAGE gels and was transferred onto a PVDF membrane. Then, the membrane was blocked with 5% skim for 30 min and incubated with primary antibody at 4 °C overnight. The working concentrations of the antibodies were anti-Mecp2 (1:1000), anti-PSD95 (1:2000), anti-TrkB (1:1000), anti-BDNF (1:2000), anti-CREB1 (1:2000), anti-p-CREB1 (1:2000), and anti-synaptophysin (1:2000). The peroxidase affinipure goat anti-rabbit or anti-mouse IgG were used as the secondary antibody to incubate at room temperature for 2 h, followed by ECL development [33]. The relative protein levels were calculated by quantification of band intensity with Image J 1.53a software (NIH, New York, NY, USA) and normalized to Tubulin, GAPDH, or β-actin.

### 2.6. Hematoxylin-Eosin (HE), Immunohistochemistry (IHC), and Nissl Staining

Brains and DRG were collected from perfused mice and subsequently stored in 4% paraformaldehyde for 24 h. For HE staining, brains were dehydrated with ethanol and subsequently embedded in paraffin. Each tissue was sectioned into 8 µm slices and stained with hematoxylin and eosin or toluidine blue stain.

The distributions of Mecp2 in DRG were detected by IHC, which was carried out using formalin-fixed paraffin-embedded tissue according to the published methods [34]. The anti-Mecp2 antibody (the working concentration 1:500) was visualized by the Vector Laboratories ImmPRESS Detection kit (Newark, NJ, USA, Cat# MP-7451 and MP-7452), which employed a second antibody conjugated with horseradish peroxidase and a diaminobenzidine-based stain. All sections were counterstained with Mayer’s hematoxylin.

Images were captured under a light microscope (Olympus BX41, Olympus Inc., Shanghai, China). Image Pro Plus 6.0 software (Media Cybernetics, Inc., Rockville, MD, USA) was used to analyze the stained neurons of Nissl images and IHC images. Average optical density (AOD) was used to evaluate the protein expression level of Mecp2. The hippocampal cell count of three mice in each group were analyzed. For each mouse, three microscope fields were randomly selected to calculate the average number of cells per square millimeter in each area of the hippocampus, including CA1, CA2, CA3, and DG.

### 2.7. Golgi Staining

Mouse brain was harvested and placed in Golgi-staining fixation solution immediately [35]. The whole brain tissue of the mouse was gently rinsed several times with normal saline, then transferred into a round-bottom centrifuge tube with 50 mL Golgi dye (Servicebio, Wuhan, China) for 14 days. Followed by washing with distilled water several times, the brain tissue was glued on the tray of the vibrating microtome with 502 super glue and immersed in pure water. Then, the brain was sectioned at 100 μm, drying at 4° overnight. After soaking the slices in concentrated ammonia water for 10 min, all slides were immersed in acid-hardening fixing bath for 45 min, and we washed the slides twice in pure water, followed by sealing it with glycerine gelatin. Digital scanning under white light and CaseViewer2.4 browsing software were performed to scan, capture, and analyze the composite image.

According to the method of Yang Y et al. [36], images were coded, and the dendritic spines from at least 20 pyramidal neurons per group in hippocampus were counted in a double-blind manner using Image J software. For Scholl analysis, at least 5 pyramidal neurons in the hippocampus were used for each group.

### 2.8. Statistical Analysis

All data are presented as mean ± SEM unless otherwise specified. Statistical analyses were determined by unpaired or paired (where applicable) Student’s *t*-test, and two-tailed *t*-test with a 95% confidence interval. Variance was analyzed by using an F-test. Where appropriate, one-way or two-way ANOVA and Tukey’s multiple comparisons test were performed. All statistical tests were performed with GraphPad Prism V8 software (Graphpad Software Inc., Boston, MA, USA). *p* value of <0.05 was considered to be statistically significant.

## 3. Results

### 3.1. Mecp2 Deficiency in Peripheral Sensory Neuron Modifies Superficial Sensation in Mice

**Figure 1:**
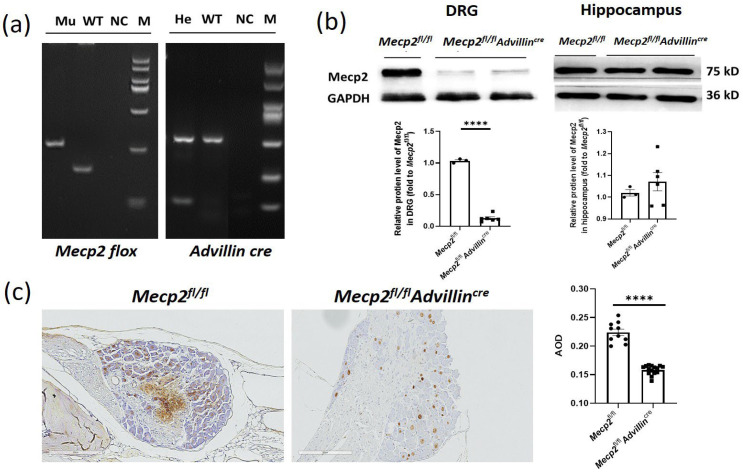
Generation of *Mecp2^fl/fl^ Advillin^cre^* mice. Female *Mecp2* floxed mice were bred with male *Advillin-Cre* mice to generate *Mecp2^fl/fl^Advillin^cre^* mice. (**a**) Analysis of the PCR products of tail DNA from pups that were produced from crossings between *Mecp2^fl/fl^Advillin^cre^* males and females carrying both *Mecp2* floxed and *Advillin-Cre* transgene by agarose gel electrophoresis. Mu: mutant (*Mecp2* 280 bp, *Advillin^cre^* 150 bp); WT: wild-type (*Mecp2* 180 bp, *Advillin^cr^*^e^ 530 bp); NC: negative control; He: heterozygous; M: DNA ladder DL2000; (**b**) western blot analysis of protein samples prepared from DRG and hippocampus of adult *Mecp2^fl/fl^* and *Mecp2^fl/fl^Advillin^cre^* mice. *Mecp2^fl/fl^ n* = 3, *Mecp2^fl/fl^Advillin^cre^ n* = 6. (**c**) Mecp2 immunohistochemical analysis of DRG sections from adult *Mecp2^fl/fl^* and *Mecp2^fl/fl^Advillin^cre^* mice. The brownish yellow particles represent positive protein expression. Scale bar: 200 µm. AOD: Average optical density. **** *p* < 0.0001.

**Figure 2:**
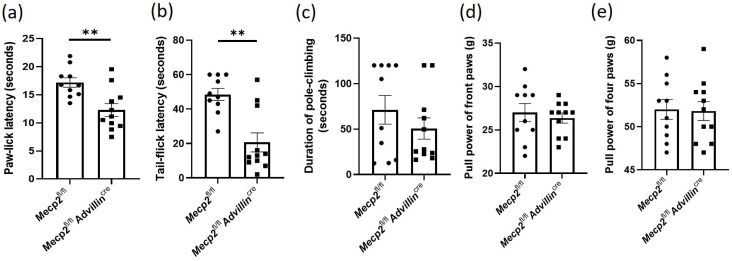
*Mecp2* deficiency in sensory neuron leads to increased sensitivity to temperature and pain. Somatic sensations of *Mecp2 ^fl/fl^* and *Mecp2^fl/fl^ Advillin^cre^* mice were measured by hot-plate test (**a**) and cold tail-flick latency reflex test (**b**). The sense of motion and position were measured by pole-climbing test (**c**), and forelimb and hindlimb grip strength (**d**,**e**). *Mecp2^fl/fl^ n* = 10, *Mecp2^fl/fl^Advillin^cre^ n* = 11. ** *p* < 0.01.

To study the in vivo function of Mecp2 in sensory neurons, *Mecp2* floxed mice were crossbred with *Advillin-Cre* mice to generate *Mecp2^fl/fl^Advillin^cre^* mice. The conditional knockout mice were confirmed with genotyping (Figure 1a). To verify the *Mecp2* expression deficiency, the protein levels in the DRG and hippocampus were assessed by western blot (Figure 1b) and immunohistochemistry (IHC) (Figure 1c). We observed a decrease in Mecp2 protein expression in the DRG, while no statistical change was observed in the hippocampus. Mice lacking the *Mecp2* gene in somatosensory neurons exhibited normal body weight, brain size, hindlimb extension, motor performance, and lifespan (Supplementary Figure S2 and Figure 2). *Mecp2^fl/fl^Advillin^cre^* mice did not show any obvious abnormalities in morphology or behavior. Thus, the *Mecp2* deletion in somatosensory neurons does not lead to overt RTT phenotypes.

We performed some tests to assess somatic sensations of *Mecp2^fl/fl^Advillin^cre^* mice, including temperature, position, and motion. Firstly, the temperature sensitivity was evaluated by cold tail-flick latency reflex test and hot-plate test. Compared to *Mecp2^fl/fl^* mice, the latencies of paw-lick and tail-flick in *Mecp2^fl/fl^Advillin^cre^* mice were shortened by 22.43% (Figure 2a) and 45.77%, respectively (Figure 2b). We next evaluated the motion function of *Mecp2*-deficiency mice by the pole-climbing test (Figure 2c) and grip strength test (Figure 2d,e). The results showed that there was no significant difference between the WT mice and *Mecp2* conditional knockout mice. Taken together, these results suggest that the deletion of *Mecp2* in the sensory neuron increases the sensitivity to temperature, but does not affect the sense of motion and position.

### 3.2. Lack of Mecp2 in Peripheral Sensory Neuron Does Not Affect Social Behaviors, Exploratory Activities, and Anxiety-like Behavior in Mice

**Figure 3:**
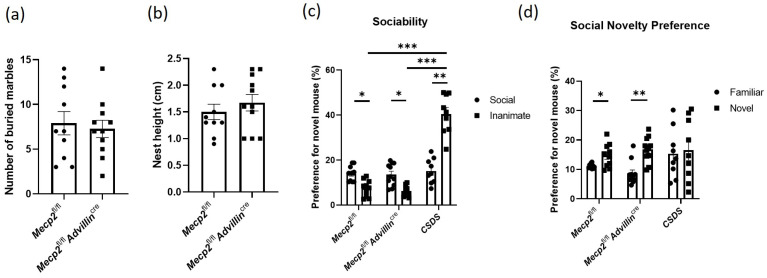
*Mecp2* in peripheral sensory neuron is not associated with compulsive-like behaviors and sociability of mice. The compulsive-like behaviors of *Mecp2^fl/fl^* and *Mecp2^fl/fl^Advillin^cre^* mice were measured by marble-burying test (**a**) and nestlet-shredding test (**b**). The sociability (**c**) and social novelty preference (**d**) of *Mecp2^fl/fl^* and *Mecp2^fl/fl^ Advillin^cre^* mice were evaluated by 3-chamber social interaction assay. *Mecp2^fl/fl^ n* = 10, *Mecp2^fl/fl^Advillin^cre^ n* = 11, CSDS *n* = 9. * *p* < 0.05, ** *p* < 0.01, *** *p* < 0.001.

We used the marble-burying test and nestlet-shredding test to assess repetitive, compulsive-like behaviors in mice. Both *Mecp2^fl/fl^* mice and *Mecp2^fl/fl^Advillin^cre^* mice buried a similar number of marbles (Figure 3a). The result of the nestlet shred test showed there was no significant difference in the nest height between *Mecp2^fl/fl^* mice and *Mecp2^fl/fl^Advillin^cre^* mice (Figure 3b). Thus, these results suggested that Mecp2 in peripheral sensory neuron is not associated with compulsive-like behaviors in mice.

The three-chamber social interaction assay is commonly used to assess both sociability and social recognition/preference in rodents, and it is often used to measure social behavior deficits in mice [37]. Here, it was used to evaluate the effect of *Mecp2* in the sensory neuron on social behaviors, and CSDS mice were simultaneously used as the positive control. We found there was no obvious abnormality in *Mecp2^fl/fl^Advillin^cre^* mice during the sociability phase. Compared to *Mecp2^fl/fl^* mice, *Mecp2^fl/fl^Advillin^cre^* mice exhibited a similar duration in the chamber containing the social target or an inanimate target (Figure 3c). During the social novelty phase, *Mecp2^fl/fl^* mice and *Mecp2^fl/fl^Advillin^cre^* mice also spent a similar time in the chamber with a familiar mouse or an unfamiliar mouse. But the CSDS mice had more time with an inanimate object during the sociability phase (Figure 3d). Interestingly, during the “social novelty preference” session, the preference for the novel mouse increased by 31.19% compared to familiar mice in *Mecp2^fl/fl^* mice, while *Mecp2^fl/fl^ Advillin^cre^* mice increased by 95.11%. It seems that *Mecp2^fl/fl^Advillin^cre^* mice displayed a strong preference for the novel mouse.

**Figure 4:**
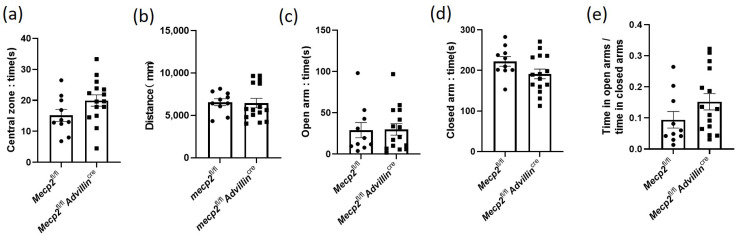
*Mecp2* in peripheral sensory neuron did not affect the anxiety-like behavior of mice. In open field test, the time spent in the central square (**a**) and the distance traveled in the arena (**b**) were measured. In elevated plus maze, the time of mice spent in the open (**c**) and closed (**d**) arms were recorded, and their ratio was calculated (**e**). *Mecp2^fl/fl^ n* = 10, *Mecp2^fl/fl^ Advillin^cre^ n* = 15.

Next, we investigated whether mice harboring or showing the deletion of *Mecp2* in the sensory neuron exhibit locomotor/exploratory activities and anxiety-like behavior. *Mecp2^fl/fl^* mice and *Mecp2^fl/fl^Advillin^cre^* mice were firstly subjected to a 10 min open field test. There was no statistically significant difference in the time in the central zone (Figure 4a) and the overall distance traveled in the open field (Figure 4b) between mutant mice and wild-type mice. Then, an additional measurement of anxiety-like behavior, the elevated plus-maze, was tested in all mice. Consistent with the results of the open field test, *Mecp2^fl/fl^* mice and *Mecp2^fl/fl^ Advillin^cre^* mice had a similar time in both open and closed arms, and there was no significant difference in the ratio of time in open arms to time in closed arms (Figure 4c–e). It is known that mice with anxiety-like behavior spend less time exploring the center of the chamber and travel shorter distances in the open field test and spent less time in the open arms during the elevated plus-maze test as well [38]. These data indicate that the lack of *Mecp2* in peripheral sensory neurons does not increase anxiety-like behavior in mice.

### 3.3. Peripheral Sensory Neuron Deletion of Mecp2 Leads to Improve Cognitive Function in Mice

**Figure 5:**
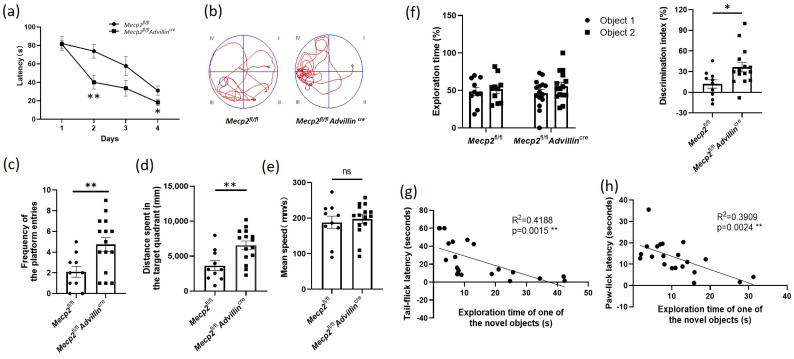
Peripheral sensory neuron deletion of *Mecp2* led to improved cognitive function in mice. Spatial learning and memory of mice were assessed by Morris water maze and object recognition test. During the training days of Morris water maze, the time it took each mouse to reach the platform (latency) was recorded (**a**). Twenty-four hours after the last training day, the probe trials of Morris water maze were performed. The activity traces (**b**), the frequency of the platform entries (**c**), the distance spent in the target quadrant (**d**), and the mean speed in the pool (**e**) were recorded. In object recognition test, during 5 min, the exploration time of one of the objects and the exploration time of the novel object were recorded and the discrimination index of objects was calculated (**f**). *Mecp2^fl/fl^ n* = 10, *Mecp2^fl/fl^Advillin^cre^ n* = 15. The inverse relationship between the exploration time of the novel object and the tail-flick latency (**g**) or paw-lick latency (**h**) were analyzed including *Mecp2^fl/fl^* and *Mecp2^fl/fl^Advillin^cre^* mice. *Mecp2^fl/fl^ n* = 10, *Mecp2^fl/fl^Advillin^cre^ n* = 11. ns: no significance, * *p* < 0.05, ** *p* < 0.01.

To learn the effect of *Mecp2* in the peripheral sensory neuron on cognitive function, we used the Morris water maze to assess the spatial learning and memory abilities of the *Mecp2* mutant and wild-type mice. During four consecutive days of training, the average latency of mice finding the escape platform in *Mecp2^fl/fl^ Advillin^cre^* mice decreased more than that in *Mecp2^fl/fl^* mice (Figure 5a). In the probe trial, we noted a 2.4-fold increase in the platform frequency of entries, and a 1.8-fold increase in the distance spent in the target quadrant of the mutant mice compared with wild-type mice (Figure 5b–d), displaying an improvement in spatial memory. There was no difference in the mean speed in the pool between the *Mecp2* mutant and wild-type mice (Figure 5e). These data suggested *Mecp2^fl/fl^Advillin^cre^* mice have better learning and memory abilities.

We also observed the difference in recognition memory between mutant and wild-type mice by the object recognition test. An increased exploration time for the novel object in 5 min was shown in *Mecp2^fl/fl^Advillin^cre^* mice compared with *Mecp2^fl/fl^* mice, but there was no difference between the mutant and wild-type mice in the exploration time of one of the objects (Sample a or b). Moreover, the DI of mutant mice was higher than that of wild-type mice (Figure 5f). These data further demonstrated *Mecp2^fl/fl^Advillin^cre^* mice have better memory ability.

To determine whether sensitivity to temperature affects the cognitive function in mice, the tail-flick latency and paw-lick latency were compared with individual performances in the object recognition test. Linear regression models were performed on the relationship between the exploration time of the novel object and the tail-flick latency or paw-lick latency. The results revealed that there was a significant interaction between the temperature sensitivity and the learning and memory abilities regardless of the mice genotype (tail-flick latency: R2 = 0.4188, *p* = 0.0015; paw-lick latency: R2 = 0.3909, *p* = 0.0024). It suggested a positive correlation between the temperature sensitivity and the cognitive performance in mice (Figure 5g,h).

### 3.4. Peripheral Sensory Neuron Deletion of Mecp2 Induces Modification Synaptic Phenotypes in Hippocampus

**Figure 6:**
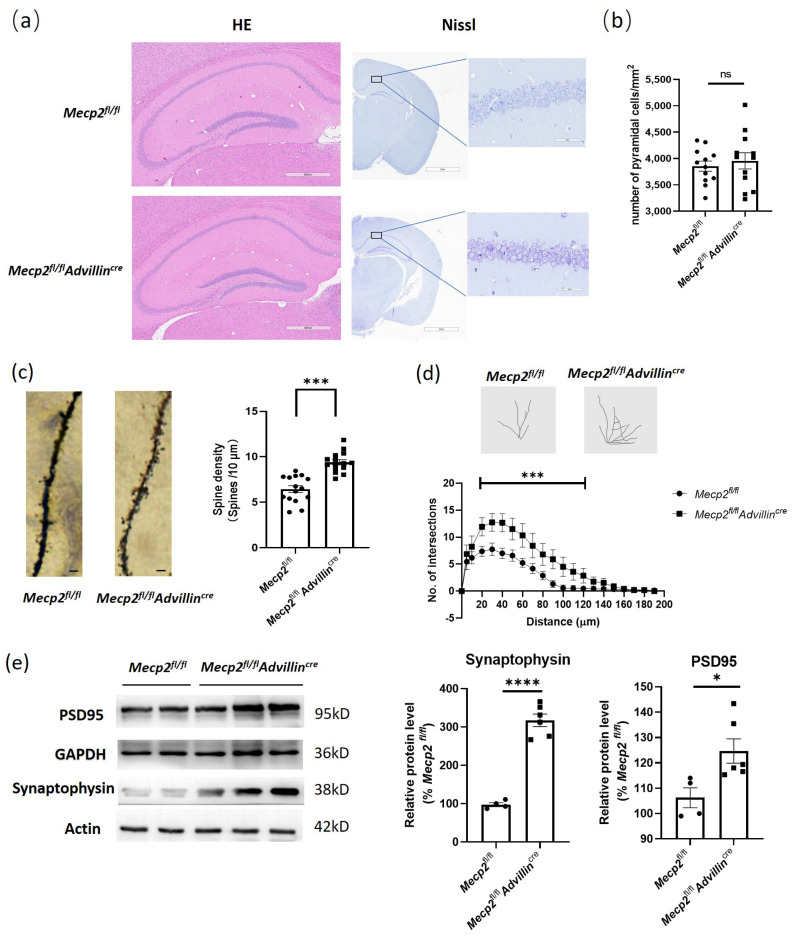
Peripheral sensory neuron deletion of *Mecp2* induced modification of synaptic phenotypes in hippocampus. Mice brains were staining by HE and toluidine blue. Images were captured under a light microscope. Scale bar: 600 µm (HE), 2 mm, and 60 µm (Nissl) (**a**). The hippocampal cell count of three mice in each group were analyzed. For each mouse, three microscope fields were randomly selected to calculate the average number of cells per square millimeter in each area of the hippocampus, including CA1, CA2, CA3, and DG (**b**). Golgi staining was performed to measure the spine density of hippocampal pyramidal neurons (**c**) and the dendritic arborization (**d**) in mice. Protein levels of PSD95 and synaptophysin were determined by western blot (**e**). The protein levels of target genes were normalized to β actin. The values for *Mecp2^fl/fl^* were set at 100% as control. *Mecp2^fl/fl^ n* = 4, *Mecp2^fl/fl^Advillin^cre^ n* = 6. ns: no significance, * *p* < 0.05, *** *p* < 0.001, **** *p* < 0.0001.

To explore the mechanisms of the cognitive function improvement in *Mecp2^fl/fl^Advillin^cre^* mice, we first used HE and Nissl staining to evaluate the brain histology and the number of neurons in the hippocampus. The results showed there was no difference between *Mecp2^fl/fl^* and *Mecp2^fl/fl^Advillin^cre^* mice (Figure 6a,b). Then, we performed Golgi staining to measure the spine density of hippocampal pyramidal neurons and found a significant increase in total spine density in *Mecp2^fl/fl^Advillin^cre^* mice compared with *Mecp2^fl/fl^* mice (Figure 6c). Of note, increased synaptic markers were concomitant with the increased dendritic arborization in *Mecp2^fl/fl^Advillin^cre^* animals (Figure 6d), indicating that, unlike the substantial reduction in dendritic trees observed in the complete knockout of *Mecp2* [39], the peripheral somatosensory neuronal-specific knockouts induced opposite changes in synaptic content and neuronal morphology. Meanwhile, the synaptophysin and PSD95 protein expression levels in the hippocampus in *Mecp2^fl/fl^Advillin^cre^* mice were higher than in *Mecp2^fl/fl^* mice (Figure 6e). These results indicate that the peripheral sensory neuron deletion of *Mecp2* induces the synaptic phenotype modification of pyramidal cells in the hippocampus.

### 3.5. Peripheral Sensory Neuron Deletion of Mecp2 Activates BDNF-TrkB-CREB1 Signaling Pathway in Hippocampus

**Figure 7:**
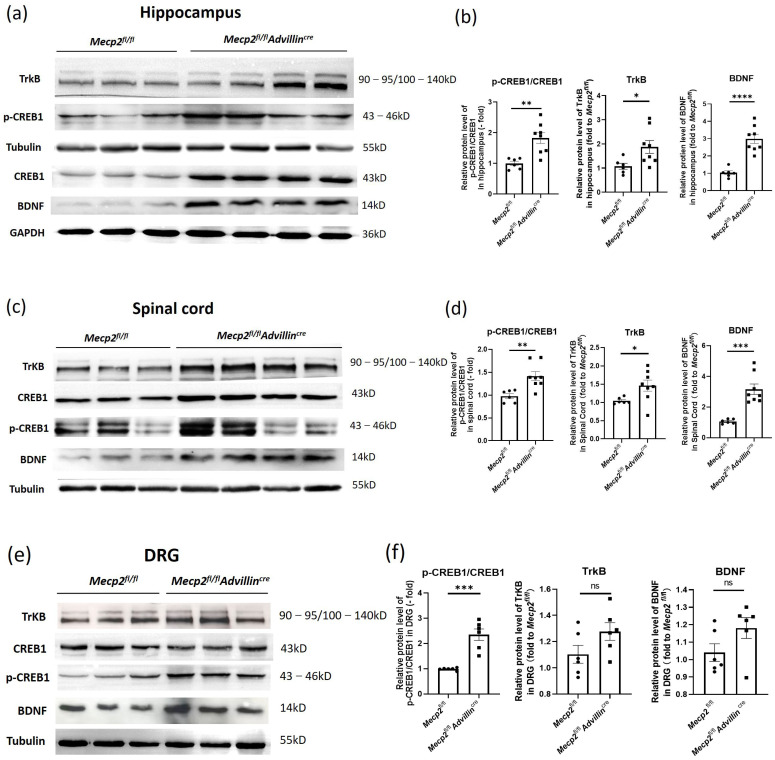
Peripheral sensory neuron deletion of *Mecp2* activates BDNF-TrkB-CREB1 signaling pathway. Protein levels of BDNF, p-CREB1, CREB1, and TrkB in hippocampus (**a**), spinal cord (**c**), and DRG (**e**) were determined by western blot. The semi-quantitative protein levels of target genes were normalized to Tubulin or GAPDH (**b**,**d**,**f**). *Mecp2^fl/fl^ n* = 6, *Mecp2^fl/fl^Advillin^cre^ n* = 8. *p* value was determined by *t*-test. ns: no significance, * *p* < 0.05, ** *p* < 0.01, *** *p* < 0.001, **** *p* < 0.0001.

To delve deeper into the neurobiological mechanisms underlying the altered synaptic phenotypes in the hippocampus of *Mecp2^fl/fl^Advillin^cre^* mice, we examined the BDNF-TrkB-CREB1 signaling pathway in the hippocampus, spinal cord, and DRG through western blot analysis. Compared to *Mecp2^fl/fl^* mice, the protein levels of BDNF, TrkB, and p-CREB1/CREB1 were significantly elevated in the hippocampus and spinal cord of *Mecp2^fl/fl^Advillin^cre^* mice (Figure 7a–d). In DRG, the ratio of p-CREB1/CREB1 significantly increased, while the TrkB and BDNF did not change (Figure 7e,f).

**Figure 8:**
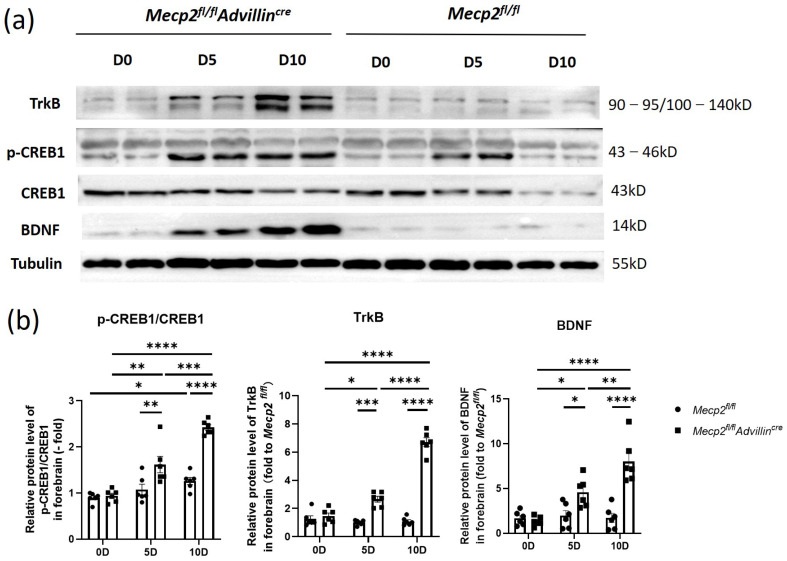
The BDNF-TrkB-CREB1 signaling pathway was activated in forebrain of *Mecp2^fl/fl^Advillin^cre^* mice after birth. Protein levels of BDNF, p-CREB1, CREB1, and TrkB in forebrain (**a**) were determined by western blot. The semi-quantitative protein levels of target genes were normalized to Tubulin (**b**). *Mecp2^fl/fl^ n* = 6, *Mecp2^fl/fl^Advillin^cre^ n* = 8. *p* value was determined by two-way ANOVA. * *p* < 0.05, ** *p* < 0.01, *** *p* < 0.001, **** *p* < 0.0001.

Next, we explored the relationship between the deletion of *Mecp2* in sensory neurons and the activation of the BDNF-TrkB-CREB1 signaling pathway in the hippocampus after mice birth. In Figure 8, the protein levels of BDNF, p-CREB1, and TrkB in *Mecp2^fl/fl^Advillin^cre^* mice were not higher than in *Mecp2^fl/fl^* mice on the date of birth. But, on the fifth day after birth, BDNF and TrkB protein expressions increased in the mutant animal, and the ratio of p-CREB1/CREB1 was elevated as well. On day 10 after birth, the expression of these proteins and the ratio of p-CREB1/CREB1 still increased. Compared to WT mice, all of these proteins in the hippocampus remained at higher levels in mutant mice even in adulthood. The results showed the activation of the hippocampal BDNF-TrkB-CREB1 signaling pathway in mutant mice began within 5 days after birth, which was earlier than that in wild-type mice. It suggests that an *Mecp2* deficiency in peripheral sensory nerves affects the environmental stimuli transmission and modulates the signal pathway of hippocampal neurons.

## 4. Discussion

There is growing evidence that peripheral mechanisms may contribute to some of the core symptoms and common comorbidities observed in neuropsychiatric disorders. For instance, mutations in genes such as *Mecp2*, *Fmr1*, and *Shank3* in PNS have been linked to core symptoms and comorbidities associated with ASD [40]. While aberrant sensory reactivity is now regarded as a diagnostic feature of ASD [13], the organization and implications of *Mecp2* involvement in terms of PNS and its effects on brain function are still not well-understood [41]. Moreover, it is unclear whether *Mecp2* in the PNS exerts similar effects in other neurological and psychiatric disorders. In the present study, we investigated the connections between somatosensory reactivity and cognitive function improvement in mice lacking *Mecp2* in the PNS ganglia. Our findings suggest that the mechanisms may be associated with the involvement of peripheral *Mecp2* in the regulation of the BDNF/CREB1 signaling pathway and the enhancement of hippocampal dendritic spine plasticity.

*Mecp2* regulates various types of peripheral neurons. In zebrafish, *Mecp2* regulates the projections of sensory neurons and sensory responses by directly activating the transcription of specific axon guidance cues, such as *Sema5b* and *Robo2* [42]. In mice, mutations of *Mecp2* and *Shank3* genes in PNS induced tactile abnormalities [13]. *Mecp2* knockdown specifically in DRG neurons increases DRG axon outgrowth and causes mechanical hypersensitivity in rats [43]. Considering that the peripheral nerves primarily control somatic sensation and movement, we evaluated the peripheral sensory function and estimated the motor ability of all mice in our study. Our data demonstrated an increased susceptibility to peripheral temperature sensory in *Mecp2^fl/fl^Advillin^cre^* mice, while the grip strength, co-ordination, and crawling ability of these animals remained unchanged. This suggests that *Mecp2* deficiency in PNS sensory neurons induces sensory hypersensitivity without affecting motor abilities.

It is known that the mutation of *Mecp2* in CNS leads to anxiety and abnormal social behaviors. However, in our study, the results of the marble-burying test and nestlet-shredding test, which assess obsessive behaviors, did not reveal significant differences between mutant mice and control mice. This suggests that stereotyped behaviors, a characteristic symptom of Autism Spectrum Disorder (ASD), may be unrelated to *Mecp2* in peripheral sensory neurons. Surprisingly, our data did not indicate that *Mecp2* knockout in peripheral sensory neurons affects anxiety-like behavior and social abilities in mice. This finding contradicts the report by Orefice et al. [8]. Several factors may contribute to this discrepancy: (1) Differences in the knockout sites of the *Mecp2* gene in the floxed mice. Despite using the same Advillin-Cre mice for constructing our experimental subjects, variations at the knockout sites may influence the outcomes. (2) Discrepancies in the extent of *Mecp2* gene deletion in tissues [44,45]. According to the information on the Jackson Laboratory website for the *Advillin-Cre* strain (032536), only heterozygous males are recommended for sensory-neuron-specific Cre recombinase expression. It is noteworthy that David Ginty’s previous studies [13,14] included both male and female *Mecp2^fl/fl^ Advillin^cre^* mice. Given our uncertainty about consistent gene deletion across all female *Mecp2^fl/fl^ Advillin^cre^* mice, our study was restricted to heterozygous male mice. Therefore, the degree of *Mecp2* deficiency in tissues of *Mecp2^fl/fl^Advillin^cre^* animals in our work and David Ginty’s may vary. (3) Sensitivity of neurotransmission regulation to *Mecp2* levels. *Mecp2*, a crucial component of neuronal chromatin, can act as both a transcriptional repressor and activator depending on the molecular context [46].

In the present work, the targeted disruption of *Mecp2* specifically in peripheral sensory neurons did not alter anxiety-like behavior or locomotion abilities in mice, enabling a focused examination of hippocampus-dependent memory performance. Interestingly, our results showed that *Mecp2^fl/f^Advillin^cre^* mice had better learning and memory abilities than *Mecp2^fl/fl^* mice. We speculate that the environmental stimuli transmitted through peripheral sensory nerves may modulate the structure and function of hippocampal neurons. Growing evidence supports the significance of environmental stimulation in shaping and refining neuronal circuits, particularly during early childhood. Both *Mecp2*-deficient mice and *Mecp2*-overexpression mice have displayed reduced spontaneous synaptic transmission as well as reduced synaptic formation, learning, and memory. Thus, *Mecp2* may control neuronal plasticity, dendritic morphology, processes associated with short- and long-term plasticity, and the balance between excitatory and inhibitory neurotransmission during the postnatal stage by regulating stimulus-dependent gene transcription [47]. Our Golgi staining results supported an increase in the dendritic complexity of hippocampal neurons in *Mecp2^fl/f^Advillin^cre^* mice, and western blot results proved the synaptophysin and PSD95 protein levels in the hippocampus were also elevated. Collectively, our findings suggest that *Mecp2* in peripheral sensory neurons subtly maintains a delicate balance in related behaviors and neural phenotypes.

Synapse formation and maturation are known to require activity-dependent gene expression in neurons. BDNF, as a secreted protein, modulates the shape and number of dendritic spines, promoting many aspects of experience-dependent synaptic development. Some studies have identified *BDNF* as a *Mecp2* target gene and abnormal levels of *Mecp2* will result in the misregulation of it [48,49]. Consistently, our data in this work demonstrated that the loss of *Mecp2* in peripheral neurons increased spinal and hippocampal BDNF expression. Meanwhile, the expression level of CREB1 of spinal and hippocampal neurons in *Mecp2^fl/fl^Advillin^cre^* mice was higher than *Mecp2^fl/fl^* mice as well. Although recent reports have suggested crosstalk between Mecp2 and BDNF/CREB in the brain [47,50,51], whether the interactions occur in peripheral somatosensory neurons or how it influences brain function remains unknown. Xian et al. found that the crosstalk between the somatosensory and sympathetic nervous systems at the DRG level contributes to the development of peripheral sensory hypersensitivity through BDNF signaling [52]. Our results show, compared to *Mecp2^fl/fl^* mice, the protein levels of BDNF, TrkB, CREB1, and p-CREB1 were significantly elevated in the hippocampus and spinal cord of *Mecp2^fl/fl^Advillin^cre^* mice. Our findings lead us to propose, in peripheral neurons, an *Mecp2* deficiency improves BDNF expression, and the BDNF expression increasing in the hippocampus contributes to synapse formation and maturation.

Some researchers believe that impairments in multiple sensory systems during critical developmental windows may affect language acquisition, cognition, anxiety, and abnormal social behaviors [53]. Hertenstein et al. found that early childhood tactile experiences were critical for the acquisition of normal social behavior and communication skills in humans and rodents [14]. We note that, in the present work, the specific deficiency of *Mecp2* in peripheral-sensory-neuron-induced related hypersensitivity responses increased the postnatal activation of the BDNF-TrkB-CREB1 signaling pathway, and improved cognition in adult mice, revealing that the superficial sensation of temperature might play important roles in cognitive development as well. Meanwhile, it suggests the rational therapeutic interventions of Mecp2 expression in peripheral sensory neurons or pharmacological strategies targeting the BDNF-TrkB-CREB1 signaling pathway can be used to treat cognition-impairment-related diseases.

## 5. Conclusions

In summary, we generated *Mecp2^fl/fl^Advillin^cre^* mice with the sensory-neuron-specific deletion of the *Mecp2* gene and found knockout *Mecp2* in PNS neurons improve the learning and memory abilities in mice. And the hippocampal synaptophysin and PSD95 protein expression levels in *Mecp2^fl/fl^Advillin^cre^* mice are higher than the control mice. In addition, the spine density of hippocampal pyramidal neurons increases in total spine density and dendritic arborization in *Mecp2^fl/fl^Advillin^cre^* mice compared to *Mecp2^fl/fl^* mice. The mechanism is associated with the activation of the BDNF-TrkB- CREB1 signaling pathway in the hippocampus and spinal cord. It suggests a potential therapeutic strategy targeting peripheral somasensory neurons to treat learning and cognitive deficits of related diseases, such as RTT, MDS, and ASD.

## Data Availability

Data are contained within the article and Supplementary Materials.

## Supplementary Materials

The following supporting information can be downloaded at: https://www.mdpi.com/article/10.3390/cells13110988/s1, Figure S1: Schematic of the sequence of behavioral tasks and histochemical analysis; Figure S2: *Mecp2*-deficiency in peripheral sensory neuron in mice does not affect hair color, brain appearance, body size and weight.
