# Supplementary material for: Mecp2 Deficiency in Peripheral Sensory Neuron Improves Cognitive Function by Enhancing Hippocampal Dendritic Spine Densities in Mice
Source: Cells. 2024 Jun 6;13(11):988. doi: 10.3390/cells13110988 (PMC11171598; doi:10.3390/cells13110988)
Supplement: Supplementary file 1 [file cells-13-00988-s001.zip › cells-2986247-supplementary.pdf]

Supplementary

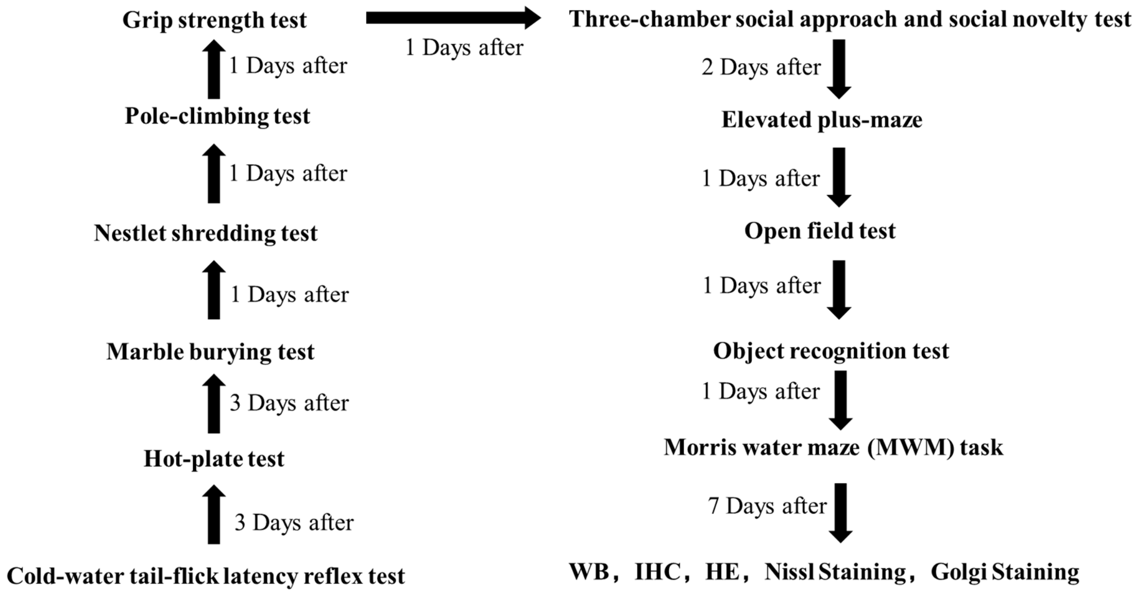

Figure S1. Schematic of the sequence of behavioral tasks and histochemical analyses.

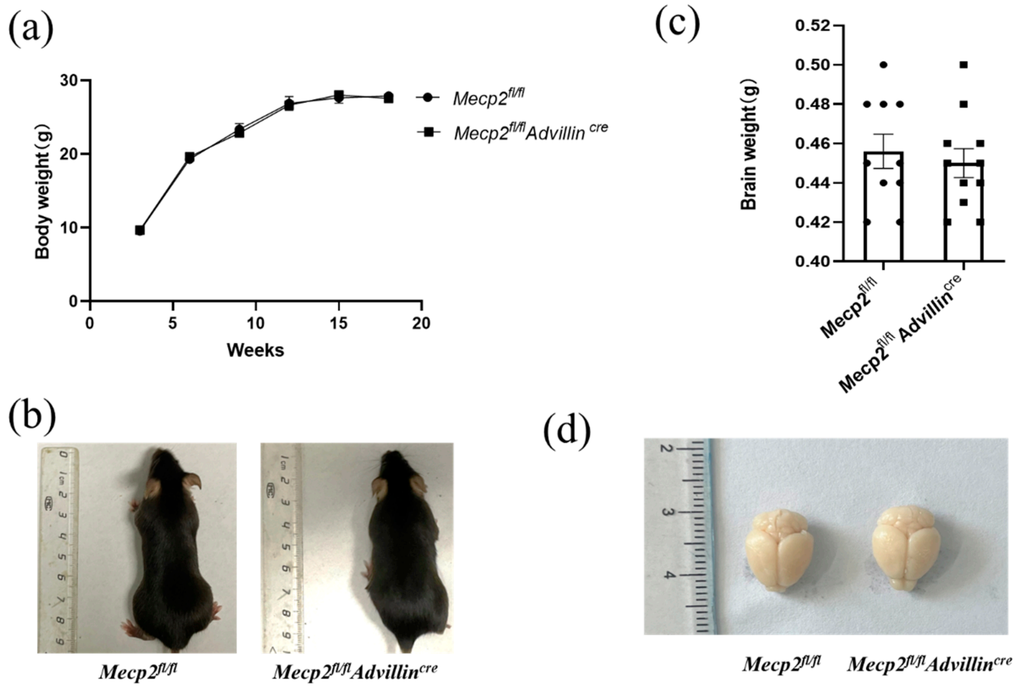

Figure S2. ***Mecp2*-deficiency in peripheral sensory neuron in mice does not affect hair color, brain appearance, body size and weight.** The body weight of mice was recorded on week 3, 6, 9, 12, 15 and 18 (a) . The image of ten-week-old mice(b). The brain was weighted at the endpoint of the experiment (c). The whole-brain image of ten-week-old mice (d).
